# Network Toxicology Reveals the Mechanisms of the Plasticizer Metabolite MECPP in Metabolic Diseases

**DOI:** 10.3390/ijms27083550

**Published:** 2026-04-16

**Authors:** Jiaqi Qiu, Chang Cheng, Biao Jiang, Diqi Yang, Hui Peng

**Affiliations:** School of Tropical Agriculture and Forestry, Hainan University, Haikou 570228, China; 15975336670@163.com (J.Q.); chengchang@hainanu.edu.cn (C.C.); jiang153018@163.com (B.J.)

**Keywords:** MECPP, network toxicology, molecular docking, metabolic disorders, phthalate exposure, environmental toxicology

## Abstract

The degradation of plastic waste leads to the release of numerous chemical additives, including phthalate plasticizers, which have been implicated in the pathogenesis of metabolic disorders. Di (2-ethylhexyl) phthalate (DEHP) is a widely used plasticizer whose primary metabolite, mono (2-ethyl-5-carboxypentyl) phthalate (MECPP), has been associated with multiple metabolic diseases. In this study, we applied an integrated approach combining network toxicology and molecular docking to systematically investigate the potential mechanistic role of MECPP in metabolic dysregulation. Our strategy included multi-platform target prediction, disease gene association analysis, functional enrichment, protein–protein interaction network construction, and molecular docking analysis. The results suggested that MECPP may be associated with six common core targets, including BCL2, BCL2L1, MAPK14, MMP2, MMP9, and TNFRSF1A, which are mainly involved in apoptosis, inflammatory regulation, and extracellular matrix remodeling. Pathway enrichment analysis further indicated the potential involvement of several disease-overlapping pathways, including insulin resistance, neuroactive ligand–receptor interaction, efferocytosis, advanced glycation end product–receptor for advanced glycation end product (AGE–RAGE) signaling, phospholipase D signaling, and renin secretion. Overall, these findings suggest that MECPP may contribute to metabolic dysregulation through overlapping molecular mechanisms across multiple diseases. This study provides a computational basis for future experimental validation and environmental risk assessment.

## 1. Introduction

Mono (2-ethyl-5-carboxypentyl) phthalate (MECPP), the primary oxidative metabolite of di (2-ethylhexyl) phthalate (DEHP), has been identified as a persistent environmental contaminant with documented endocrine-disrupting effects in both humans and animals. As the major metabolite of the plasticizer DEHP, MECPP primarily originates from polyvinyl chloride (PVC) products, including medical devices, food packaging, and consumer goods, from which it readily leaches into environmental matrices such as indoor air, household dust, and aquatic systems [1]. Biomonitoring studies consistently detect MECPP in biological samples, confirming its systemic distribution and persistence across tissues in exposed populations. Recent research raises particular concern regarding the compound’s bioaccumulation potential and its capacity to interfere with metabolic processes, as evidenced across multiple exposure pathways that contribute to measurable internal doses reflecting both recent exposures and cumulative body burden [2,3].

Epidemiological studies consistently link MECPP exposure to a 16% increase in metabolic syndrome risk across populations, reflecting its capacity to disrupt critical metabolic signaling pathways [4]. The compound’s environmental persistence and bioaccumulative properties likely exacerbate these effects through chronic exposure, with computational models identifying MECPP interactions with key regulatory nodes in metabolic networks that may underlie observed associations between phthalates and metabolic syndrome. Atherosclerosis, diabetes, hypertension, and nonalcoholic fatty liver disease (NAFLD) share fundamental pathological links through insulin resistance and chronic inflammation. These disorders exhibit bidirectional relationships: diabetes exacerbates atherosclerosis through hyperglycemia-induced oxidative stress, whereas hypertension accelerates vascular damage and plaque formation [5,6,7]. NAFLD functions both as a manifestation and an amplifier of metabolic dysfunction, with disease progression from steatosis to fibrosis paralleling increased cardiovascular risk [8,9]. As a primary DEHP metabolite, MECPP possesses structural characteristics that facilitate nuclear receptor interactions, particularly disruption of the peroxisome proliferator–activated receptor (PPAR) pathway, potentially explaining its capacity to influence this interconnected disease network through environmental exposure [10].

Network toxicology integrates multi-omics data to construct comprehensive interaction networks among compounds, targets, and pathways, thereby providing a systems-level framework for analyzing complex toxicological mechanisms [11]. This approach effectively maps protein–protein interactions and predicts molecular pathways through which environmental toxicants, such as MECPP, may contribute to disease development [12]. Building upon these network-based predictions, molecular docking can further characterize potential interactions between small molecules and biological targets at the structural level, thereby offering preliminary insight into possible ligand–target binding patterns and affinities [13].

This study was designed as a preliminary computational exploratory analysis integrating network toxicology and molecular docking to identify and prioritize potential targets and pathways associated with MECPP across four metabolic diseases, including atherosclerosis, diabetes mellitus, hypertension, and NAFLD. By combining protein–protein interaction analysis, topological screening, enrichment analysis, and molecular docking, the present study aimed to identify biologically relevant targets and pathways, generate testable hypotheses regarding the toxicological mechanisms of MECPP, and provide a rational basis for subsequent hypothesis-driven experimental validation.

## 2. Results

### 2.1. Identification of MECPP-Related Targets Associated with Metabolic Diseases

**Figure 1:**
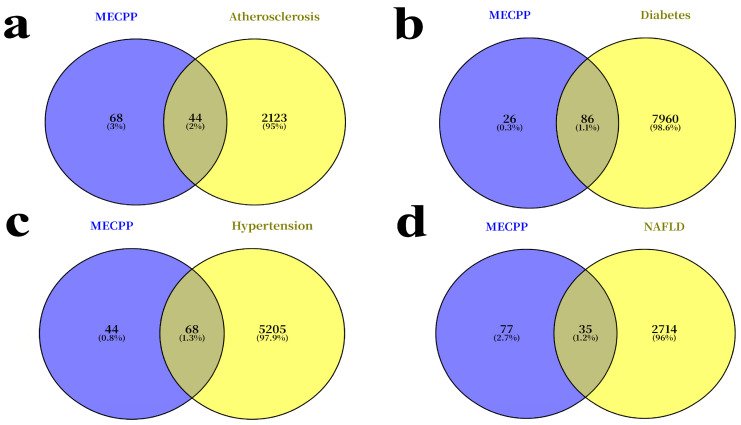
Venn diagrams showing overlapping targets between MECPP and four metabolic diseases. (**a**) Overlapping targets between MECPP and atherosclerosis; (**b**) overlapping targets between MECPP and diabetes mellitus; (**c**) overlapping targets between MECPP and hypertension; (**d**) overlapping targets between MECPP and NAFLD.

Potential MECPP-associated targets were collected through a multi-database strategy using SwissTargetPrediction, ChEMBL, and STITCH. SwissTargetPrediction returned 100 candidate targets, with a probability score of 0.1157, whereas STITCH and ChEMBL provided additional interaction-supported target information. After integration, deduplication, and UniProt-based standardization, 112 unique MECPP-associated targets were retained for downstream analysis. Disease-related targets were collected from GeneCards (relevance score > 1) and OMIM, yielding 2170 for atherosclerosis, 8107 for diabetes mellitus, 5298 for hypertension, and 2674 for nonalcoholic fatty liver disease (NAFLD). Comparative analysis using Venn diagrams revealed 44, 86, 68, and 35 overlapping targets between MECPP and the respective disease-associated gene sets (Figure 1a–d; Table S1, Supporting Information). The resulting changes in disease-target set size, MECPP–disease overlap, and common core target stability under alternative threshold settings are summarized in (Tables S11 and S12, Supporting Information). These intersections suggest that MECPP may perturb multiple molecular processes relevant to vascular, endocrine, and hepatic metabolic dysfunction.

### 2.2. Construction and Analysis of the PPI Network Associated with MECPP and Metabolic Diseases

Protein–protein interaction (PPI) networks for the overlapping targets between MECPP and each metabolic disease are constructed using the STRING database and visualized in Cytoscape 3.10.1.

**Figure 2:**
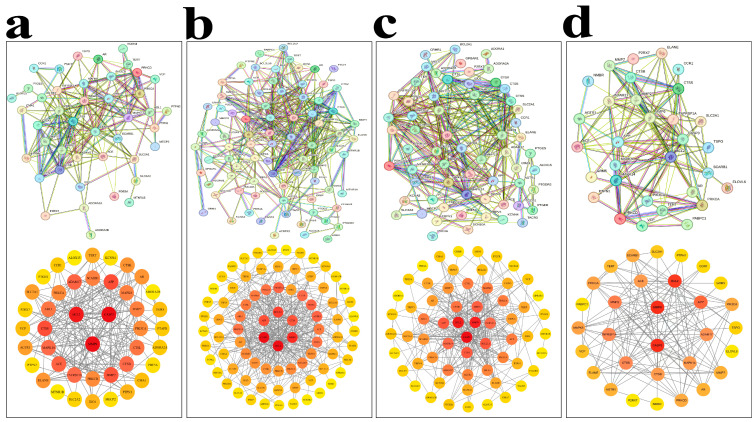
Protein–protein interaction (PPI) network analysis of overlapping targets between MECPP and four metabolic diseases. Panels (**a**–**d**) correspond to atherosclerosis, diabetes mellitus, hyper-tension, and NAFLD, respectively. In each panel, the upper subpanel shows the PPI network constructed using the STRING database and visualized in Cytoscape, whereas the lower subpanel shows the top 10 hub genes ranked by degree centrality. In the PPI networks, nodes represent target proteins and edges represent protein–protein interactions. Darker node colors indicate higher degree centrality.

The network for atherosclerosis contains 43 nodes and 193 edges, with an average degree of 8.98, a diameter of 4, a radius of 1, a characteristic path length of 1.737, and a clustering coefficient of 0.249 (Figure 2a). The color intensity of nodes reflects target relevance to atherosclerosis pathogenesis. The diabetes-related network consists of 81 nodes and 358 edges, where nodal color intensity represents target relevance to diabetes-associated mechanisms (Figure 2b). For hypertension, STRING-based analysis generated a PPI network comprising 62 nodes and 280 edges, with a network diameter of 5, characteristic path length of 1.987, clustering coefficient of 0.004, and network density of 0.068 (Figure 2c). Collectively, these topological parameters indicate that the network retains overall connectivity but exhibits minimal local clustering, consistent with a relatively sparse and weakly modular organization rather than a highly clustered structure. The NAFLD-associated network included 31 nodes and 139 edges, with an average degree of 8.97, network diameter of 4, characteristic path length of 1.598, clustering coefficient of 0.278, and density of 0.149, suggesting a moderately modular topology with small-world characteristics (Figure 2d).

### 2.3. Enrichment Analyses for GO and KEGG

**Figure 3:**
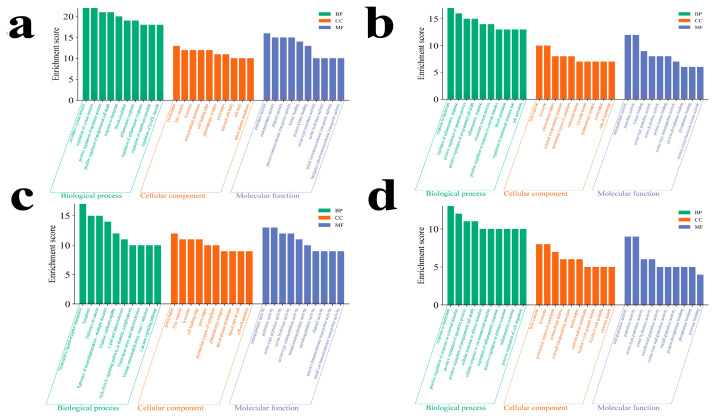
Gene Ontology (GO) enrichment analysis of overlapping targets between MECPP and four metabolic diseases. Panels (**a**–**d**) correspond to atherosclerosis, diabetes mellitus, hypertension, and NAFLD, respectively. For each disease, enriched GO terms are grouped into biological process (BP), cellular component (CC), and molecular function (MF) categories. Bar height represents the enrichment score, and different colors indicate the three GO categories. In each panel, the top 10 significantly enriched GO terms are displayed separately for BP, CC, and MF.

**Figure 4:**
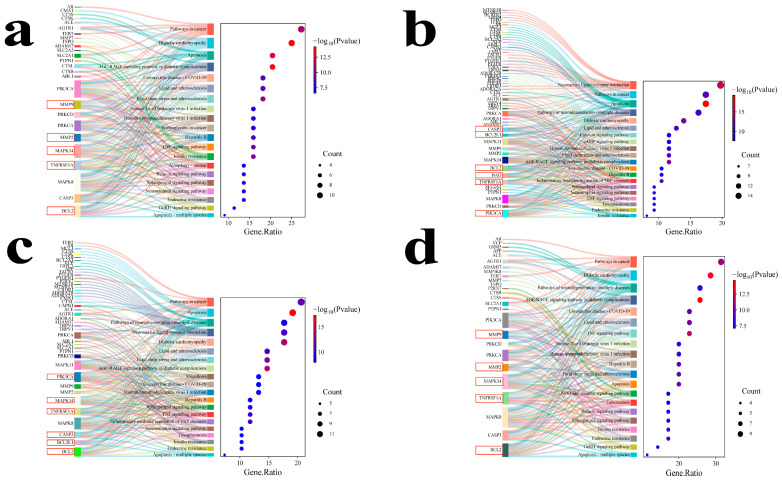
KEGG pathway enrichment analysis of overlapping targets between MECPP and four metabolic diseases. Panels (**a**–**d**) correspond to atherosclerosis, diabetes mellitus, hypertension, and NAFLD, respectively. In each panel, the left subpanel shows a gene–pathway Sankey diagram il-lustrating the relationships between representative target genes and enriched KEGG pathways, whereas the right subpanel shows the corresponding dot plot of enriched pathways. In the dot plots, dot color indicates −log10 (*p* value) and dot size indicates the number of genes enriched in each pathway. Red boxes highlight representative genes discussed in the main text. Only the top 20 significantly enriched KEGG pathways are shown.

Functional enrichment analyses are performed using the Metascape database (*Homo sapiens*) to characterize the biological significance of the overlapping targets between MECPP and each metabolic disease. The atherosclerosis-related targets yield 205 KEGG pathways and 3771 Gene Ontology (GO) terms, including 3022 biological processes (BPs), 298 cellular components (CCs), and 451 molecular functions (MFs). Enriched BP terms primarily involve the regulation of inflammatory response (FDR = 2.64 × 10^−14^), response to bacterium (FDR = 1.01 × 10^−12^), and positive regulation of apoptotic process (FDR = 7.10 × 10^−12^). CC and MF categories are notably enriched in the endosome lumen (FDR = 2.02 × 10^−10^) and endopeptidase activity (FDR = 2.25 × 10^−9^), respectively. KEGG analysis highlights diabetic cardiomyopathy (FDR = 2.44 × 10^−12^), AGE–RAGE signaling (FDR = 4.21 × 10^−12^), and apoptosis (FDR = 4.63 × 10^−11^) (Figure 3a and Figure 4a; Table S2, Supporting Information).

The diabetes-associated targets show enrichment in 218 KEGG pathways and 4431 GO terms, including 3489 BP, 361 CC, and 581 MF. Key BP terms include circulatory system process (FDR = 1.75 × 10^−15^) and positive regulation of apoptosis (FDR = 3.42 × 10^−14^). CC enrichment involves the endosome lumen (FDR = 2.73 × 10^−8^) and Bcl-2 family protein complex (FDR = 4.08 × 10^−8^), while MF enrichment emphasizes endopeptidase activity (FDR = 3.19 × 10^−9^) and channel activity (FDR = 2.22 × 10^−8^). KEGG pathways are dominated by apoptosis (FDR = 1.91 × 10^−17^), neuroactive ligand–receptor interaction (FDR = 7.20 × 10^−14^), and AGE–RAGE signaling in diabetic complications (FDR = 3.81 × 10^−11^) (Figure 3b and Figure 4b; Table S3, Supporting Information).

The hypertension-related network identifies 209 KEGG pathways and 3253 BP, 349 CC, and 531 MF terms. The most enriched BP terms include apoptosis (FDR = 1.91 × 10^−17^) and neuroactive ligand–receptor interaction (FDR = 7.20 × 10^−14^). CC analysis indicates enrichment in the endolysosome lumen (FDR = 2.31 × 10^−7^) and cell leading edge (FDR = 2.50 × 10^−6^), while MF enrichment focuses on endopeptidase and peptidase activities. KEGG analysis reveals significant enrichment in apoptosis (FDR = 1.75 × 10^−15^), AGE–RAGE signaling (FDR = 4.32 × 10^−12^), and diabetic cardiomyopathy (FDR = 4.32 × 10^−12^) (Figure 3c and Figure 4c; Table S4, Supporting Information).

Similarly, the NAFLD-associated targets are enriched in 200 KEGG pathways and 3353 GO terms, including 2656 BP, 288 CC, and 409 MF. Prominent BP terms include bacterial response (FDR = 1.44 × 10^−8^) and cellular response to abiotic stimulus (FDR = 1.49 × 10^−8^). CC enrichment occurs in endosomal luminal and ficolin-1-rich granule compartments, while MF enrichment highlights peptidase and serine-type peptidase activities. KEGG analysis demonstrates strong associations with AGE–RAGE signaling (FDR = 8.59 × 10^−13^), diabetic cardiomyopathy (FDR = 4.74 × 10^−12^), and TNF signaling (FDR = 1.18 × 10^−10^) (Figure 3d and Figure 4d; Table S5, Supporting Information).

Enrichment analyses across the four metabolic diseases revealed several disease-overlapping pathway patterns. Insulin resistance, neuroactive ligand–receptor interaction, and efferocytosis were shared by atherosclerosis, hypertension, and NAFLD. Human T-cell leukemia virus 1 infection, insulin secretion, and lysosome were common to atherosclerosis and hypertension, whereas AGE–RAGE signaling pathway in diabetic complications, pathways in cancer, phospholipase D signaling pathway, and renin secretion were shared by NAFLD and hypertension. These pathway overlaps indicate that the overlapping targets of MECPP and the four metabolic diseases are connected through partially shared biological processes.

### 2.4. Screening of Core Targets

Core-target prioritization was performed using an integrated Cytoscape-based workflow combining cytoNCA, MCODE, and cytoHubba. The cytoNCA plugin was used to evaluate degree, betweenness, and closeness centrality, and nodes exceeding the corresponding median values were retained for further analysis. MCODE was applied to identify densely connected functional modules, whereas cytoHubba ranked candidate hub genes using the maximal clique centrality algorithm. To improve the transparency and interpretability of target screening, three sequential levels of prioritization were defined in this study: disease-specific prioritized targets identified within each individual disease network, candidate core targets summarized after cross-disease comparison, and final common core targets retained after strict intersection across all four diseases.

**Figure 5:**
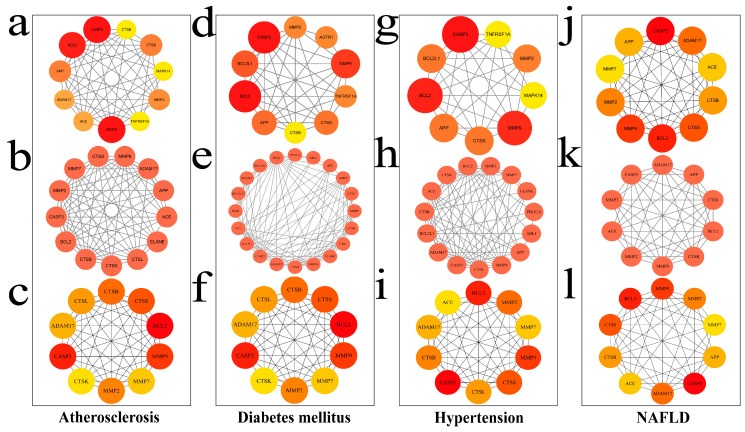
Integrated prioritization of MECPP-associated targets across four metabolic diseases. For atherosclerosis, panels (**a**–**c**) show the targets retained by cytoNCA, the functional module identified by MCODE, and the top hub genes ranked by CytoHubba, respectively. Panels (**d**–**f**), (**g**–**i**), and (**j**–**l**) show the corresponding results for diabetes mellitus, hypertension, and NAFLD, respectively. Node color reflects the relative ranking of the targets, with red indicating higher-ranked nodes and yellow indicating lower-ranked nodes. Disease-specific prioritized targets were defined as the union of targets identified by the three algorithms, and final common core targets were subsequently obtained by cross-disease intersection.

For each metabolic disease, the union of targets retained by cytoNCA, MCODE, and cytoHubba was defined as the disease-specific prioritized target set. Based on this integrated strategy, 15 disease-specific prioritized targets were identified for atherosclerosis (Table S6, Supporting Information; Figure 5a–c), 23 for diabetes mellitus (Table S7, Supporting Information; Figure 5d–f), 18 for hypertension (Table S8, Supporting Information; Figure 5g–i), and 12 for NAFLD (Table S9, Supporting Information; Figure 5j–l). Representative targets in the atherosclerosis-related set included BCL2, MAPK14, TNFRSF1A, MMP9, and MMP2. In diabetes mellitus, BCL2, BCL2L1, TNFRSF1A, CASP3, BAD, and PIK3CA were among the most prominent targets. In hypertension, BCL2, BCL2L1, MAPK14, TNFRSF1A, CASP3, and PIK3CA showed strong topological relevance. In NAFLD, BCL2, MAPK14, MMP9, MMP2, and TNFRSF1A were retained as representative prioritized targets.

Final common core targets were ultimately determined through integrated cross-disease comparison of the disease-specific prioritized target sets, together with topological relevance and biological interpretability. Based on this strategy, six common core targets were retained for subsequent analysis: BCL2, BCL2L1, MAPK14, MMP2, MMP9, and TNFRSF1A. These shared targets were also broadly consistent with the recurrent enrichment patterns observed across the four diseases.

### 2.5. The Results for Molecular Docking

Molecular docking analysis was conducted to evaluate the binding characteristics between MECPP and key protein targets associated with atherosclerosis, diabetes mellitus, hypertension, and NAFLD. All docking simulations were performed using AutoDock, and the resulting docking energies ranged from −4.68 to −5.93 kcal/mol, indicating possible ligand–protein interactions within a weak-to-moderate range (Table S10, Supporting Information).

**Figure 6:**
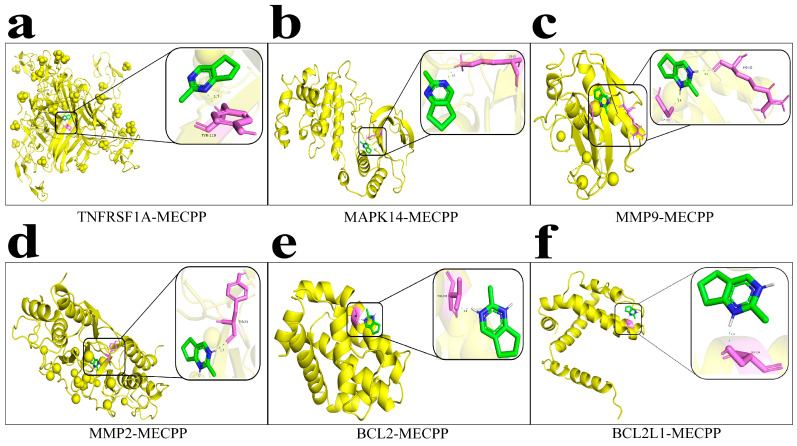
Molecular docking models showing the lowest-energy conformations of MECPP bound to representative protein targets associated with atherosclerosis, diabetes mellitus, hypertension, and NAFLD. (**a**) TNFRSF1A (−5.93 kcal/mol), (**b**) MAPK14 (−5.55 kcal/mol), (**c**) MMP9 (−5.49 kcal/mol), (**d**) MMP2 (−5.19 kcal/mol), (**e**) BCL2 (−5.38 kcal/mol), and (**f**) BCL2L1 (−5.39 kcal/mol). MECPP is shown as green/blue sticks, the interacting amino acid residues are shown as light magenta sticks, and the protein backbone is displayed as a yellow cartoon. Binding energies between −5 and −7 kcal/mol indicate weak-to-moderate predicted ligand–protein affinity. Molecular docking was carried out using AutoDock, with input preparation in AutoDockTools-1.5.7, and the docked complexes were visualized in PyMOL 3.1.5.1. Detailed docking parameters are provided in Table S10 (Supporting Information).

Several targets were commonly retained for focused analysis across the four metabolic disease models, including BCL2, BCL2L1, MAPK14, MMP2, MMP9, and TNFRSF1A. Among these, TNFRSF1A showed the lowest docking energy (−5.93 kcal/mol), with a predicted hydrogen-bonding interaction involving TYR119 (Figure 6a). MAPK14 displayed a docking energy of −5.55 kcal/mol, with the predicted complex involving the LYS53 side-chain amine group (Figure 6b). For matrix metalloproteinases, MMP9 (−5.49 kcal/mol) and MMP2 (−5.19 kcal/mol) showed predicted hydrogen-bonding interactions with GLY197, ARG162, and TYR23, respectively (Figure 6c,d).

The apoptosis-related proteins BCL2 and BCL2L1, which were retained in the cross-disease target analysis, exhibited comparable docking energies (−5.38 and −5.39 kcal/mol, respectively). The BCL2–MECPP complex showed a predicted electrostatic interaction with ARG98 (Figure 6e), whereas BCL2L1–MECPP showed a predicted hydrogen bond between the ligand HN atom and the SER154 side-chain hydroxyl group (Figure 6f). Overall, these docking results provide preliminary structural support for possible interactions between MECPP and the prioritized targets, but should be interpreted cautiously as qualitative docking predictions rather than validated measures of binding affinity.

## 3. Discussion

This study integrates network analysis with molecular docking to investigate the potential influence of MECPP on metabolic diseases, identifying shared targets across atherosclerosis, diabetes mellitus, hypertension, and NAFLD. The AGE-RAGE signaling pathway in diabetic complications (FDR = 3.81 × 10^−11^) emerged as a highly significant shared node, while additional pathways related to metabolic disorders—including insulin resistance (FDR = 5.03 × 10^−7^), sphingolipid signaling (FDR = 4.86 × 10^−8^), and diabetic cardiomyopathy (FDR = 6.11 × 10^−11^)—were also enriched. Molecular docking provided preliminary structural support for possible interactions between MECPP and proteins implicated in insulin signaling and inflammatory processes, with docking energies ranging from −4.68 to −5.93 kcal/mol. These values fall within the weak-to-moderate range and therefore should be interpreted cautiously rather than as evidence of strong target engagement. Moreover, the present docking analysis was not benchmarked against co-crystallized or reference ligands for all targets, and therefore the predicted binding energies should be interpreted only as relative structural indicators rather than validated measures of binding strength. Although these computational findings are broadly compatible with previous evidence suggesting that phthalate exposure may disrupt metabolic homeostasis, further biochemical and cellular validation is still required to determine whether such predicted interactions are biologically meaningful under environmentally relevant exposure conditions. In addition, the relevance of these predicted interactions depends on the internal dose of MECPP achieved in vivo. Reported urinary MECPP concentrations in the general population range from 3 to 45 μg/L, with levels up to 150 μg/L in occupationally exposed individuals [14,15]. However, urinary biomonitoring does not directly reflect target-tissue concentrations, and further pharmacokinetic studies are needed to clarify the internal-dose range at which MECPP may plausibly perturb these pathways.

Molecular analyses identified six common core targets—BCL2, BCL2L1, MAPK14, MMP2, MMP9, and TNFRSF1A—that may help explain how MECPP is linked to multiple metabolic diseases through partially shared but context-dependent mechanisms [16,17,18]. From a functional perspective, BCL2 and BCL2L1 are most closely related to apoptosis regulation and cell survival. This is biologically plausible because Bcl-2 family signaling has been implicated in vascular cell apoptosis during atherosclerosis, including endothelial injury, smooth muscle cell loss, and macrophage-associated plaque vulnerability, thereby linking apoptotic imbalance to lesion progression and instability [19]. MAPK14 appears to represent a stress-responsive and inflammatory signaling node. In atherosclerosis-related settings, p38α/MAPK14 is the predominant activated p38 isoform in monocytes/macrophages, colocalizes with macrophage-rich plaque regions, and is involved in eLDL-triggered inflammatory responses, cytokine secretion, and apoptosis-related signaling, supporting its role in vascular inflammatory injury [20]. TNFRSF1A, as a key receptor in TNF-mediated signaling, further strengthens the inflammatory interpretation of the present network, since its increased expression has been reported in T2DM combined with MAFLD and is associated with insulin resistance, inflammation, and hepatic lipid deposition [21].

In parallel, MMP2 and MMP9 provide a mechanistic bridge between vascular and hepatic tissue remodeling. In atherosclerosis, both enzymes are highly expressed in unstable plaques, particularly in macrophage-rich vulnerable regions, where they contribute to collagen degradation, fibrous cap weakening, and plaque instability [22,23]. This process has also been linked to inflammatory COX-2/mPGES-1/PGE2-dependent signaling and can be further influenced by hypertension-related renin–angiotensin activity, suggesting that MMP-mediated remodeling may connect atherosclerosis and hypertension at the vascular level [24]. In metabolic liver disease, MMP2 appears particularly relevant to fibrosis-related progression in NAFLD, whereas MMP9 has been associated with inflammatory and remodeling activity but may show stage-dependent or context-dependent variation across studies. Together, these observations support the interpretation that the common core targets identified here do not act uniformly across all four diseases; rather, they may converge on shared upstream processes such as apoptosis, inflammatory signaling, and extracellular matrix remodeling while contributing to different downstream pathological manifestations in vascular, hepatic, and metabolic tissues [25].

Pathway analysis further suggested that the enriched pathways may reflect disease-overlapping mechanisms rather than simple comorbidity. In particular, insulin resistance, neuroactive ligand–receptor interaction, and efferocytosis were shared by atherosclerosis, hypertension, and NAFLD, suggesting that these three conditions may converge on a common background of metabolic dysregulation, neurohumoral imbalance, and impaired resolution of cellular injury [26,27,28]. Insulin resistance is especially relevant in this context, as it has been linked not only to systemic metabolic dysfunction but also to endothelial impairment, vascular inflammation, and hepatic lipid accumulation [29,30]. Efferocytosis may likewise represent an important common theme, because defective clearance of apoptotic cells promotes persistent inflammation and tissue injury; this mechanism has been implicated in both necrotic core formation during atherosclerosis and progression from steatosis to chronic inflammatory and fibrotic injury in fatty liver disease [31,32,33].

Additional overlap was observed between atherosclerosis and hypertension, including Human T-cell leukemia virus 1 infection, insulin secretion, and lysosome pathways. These terms should not be interpreted literally as evidence of viral infection or isolated pancreatic dysfunction; rather, they more likely reflect broader modules involving inflammatory activation, endothelial injury, metabolic dysregulation, and cellular stress responses [34,35,36,37,38]. A second overlapping axis was identified between NAFLD and hypertension, in which AGE–RAGE signaling pathway in diabetic complications, pathways in cancer, phospholipase D signaling pathway, and renin secretion were shared. Among these, AGE–RAGE signaling appears particularly important because it links oxidative stress, inflammatory amplification, insulin resistance, endothelial dysfunction, and profibrotic signaling [39,40,41]. Likewise, phospholipase D signaling and renin secretion suggest a mechanistic interface between vascular regulation and hepatic metabolic stress, whereas the enrichment of “pathways in cancer” is more plausibly interpreted as reflecting aberrant proliferation, inflammatory reprogramming, anti-apoptotic adaptation, and tissue remodeling rather than literal tumorigenesis [42,43,44,45,46,47,48,49]. These pathway-level observations indicate that MECPP-associated targets are not randomly distributed across unrelated disease networks, but instead cluster within partially shared biological programs involving insulin resistance, inflammatory amplification, oxidative stress, defective clearance of dying cells, vascular remodeling, and hepatic fibrogenesis [50].

As a preliminary computational study based on network toxicology and molecular docking, the present work was intended to prioritize potentially relevant targets and pathways associated with MECPP-related metabolic disorders and to generate testable mechanistic hypotheses, rather than to provide direct experimental confirmation. Nevertheless, several limitations should be acknowledged. First, the current findings are mainly derived from computational prediction and literature-supported interpretation, and no in vitro or in vivo experiments were conducted to validate the identified targets and pathways. Second, database-related bias and annotation incompleteness may have affected target identification, particularly for less well-characterized metabolic regulators, and prediction tools may show limited accuracy for compounds with relatively unusual structural features such as MECPP [12,51,52,53]. Third, molecular dynamics simulation was not performed in the present study. Therefore, the dynamic stability of the predicted MECPP–target complexes remains to be further evaluated, and the docking results should be interpreted as preliminary structural indications rather than evidence of stable binding behavior. Fourth, the present analysis did not incorporate pharmacokinetic or internal-dose evaluation linking computational predictions to target-tissue exposure. Given that phthalate exposure occurs through ingestion, inhalation, and dermal absorption, whereas tissue distribution depends on partitioning, metabolism, and exposure route, urinary biomonitoring data alone remain insufficient to explain steady-state distribution across organs and tissues [54,55]. Recent pharmacokinetic modeling studies further suggest that plasma and urinary biomonitoring data can be integrated through reverse dosimetry to improve exposure estimation and risk assessment [56,57]. Therefore, the docking and pathway analyses presented here should be interpreted as mechanism-oriented prioritization rather than direct evidence of target-organ exposure.

Despite these limitations, the present study provides an integrated view of how MECPP may be associated with atherosclerosis, diabetes mellitus, hypertension, and NAFLD through shared yet context-dependent molecular mechanisms. From a public health perspective, the combination of widespread exposure and computational evidence of pathway-level disruption highlights the need for further investigation of MECPP-related metabolic risk [58,59]. Future studies should combine biomonitoring, pharmacokinetic or PBPK modeling, exposure-relevant in vitro and in vivo validation, and structure-based approaches such as molecular dynamics simulation to clarify exposure–response relationships and confirm the mechanistic roles of the prioritized targets at the gene and protein levels [60,61,62].

## 4. Materials and Methods

### 4.1. MECPP Composition and Target Acquisition

We first retrieved the chemical structure of MECPP and its canonical SMILES representation from the PubChem database (https://pubchem.ncbi.nlm.nih.gov/) [63]. Using the obtained SMILES representation, we queried the STITCH (http://stitch.embl.de/, accessed on 15 May 2025), SwissTargetPrediction (http://swisstargetprediction.ch/, accessed on 15 May 2025) [64], and ChEMBL databases (https://www.ebi.ac.uk/chembl/, accessed on 15 May 2025) [65], specifying *Homo sapiens* as the target species. In SwissTargetPrediction, targets with non-zero probability scores were initially retained as candidate targets. The target records obtained from STITCH, SwissTargetPrediction, and ChEMBL were then integrated and deduplicated, and the UniProt database (https://www.uniprot.org/, accessed on 15 May 2025) [66] was used to standardize target nomenclature. The final target library for MECPP was compiled after multi-database integration and deduplication.

### 4.2. Target Construction of Diseases

Disease target libraries were primarily constructed using the GeneCards (https://www.genecards.org/, accessed on 18 May 2025) [67] and OMIM (https://www.omim.org/, accessed on 18 May 2025) databases, as previously described by Stelzer et al. Keywords including “atherosclerosis,” “hypertension,” “diabetes mellitus,” and “non-alcoholic fatty liver disease” were used to retrieve disease-associated targets from these databases. To retain potentially relevant disease-associated genes while avoiding excessive inclusion of weakly related entries, the GeneCards relevance-score threshold was initially considered with reference to the median score distribution, and genes with scores above this range were examined. After comprehensive evaluation of both target quantity and target relevance, a final inclusion threshold of score > 1 was adopted for GeneCards. The GeneCards- and OMIM-derived targets were then integrated and deduplicated, yielding a final disease target library comprising 10,447 unique gene entries. To further assess the robustness of disease-target collection, an additional sensitivity analysis was performed using alternative GeneCards relevance-score thresholds (>2 and >5) for all four diseases, while OMIM-derived targets were kept unchanged.

### 4.3. The Intersection of MECPP Targets and Disease Targets

The intersection of compound targets and disease targets was identified using the Venny 2.1.0 platform (http://www.liuxiaoyuyuan.cn/, accessed on 3 August 2025). The MECPP target library and the NAFLD target library were uploaded to the platform, and the intersection results were obtained following submission.

### 4.4. Construction of Protein–Protein Interaction (PPI) Networks

The protein–protein interaction (PPI) network was constructed using the STRING database (version 11.5, https://cn.string-db.org/, accessed on 3 August 2025) [68], with *Homo sapiens* specified as the reference species and an interaction score threshold of ≥0.4. The resulting network was imported into Cytoscape (version 3.10.0) [69] for comprehensive topological analysis. To identify core genes associated with MECPP-induced metabolic disease pathogenesis, three complementary analytical approaches were employed: (1) the MCODE plugin (http://apps.cytoscape.org/apps/mcode, accessed on 3 August 2025) was applied to detect densely connected clusters using default parameters (degree cutoff = 2, node score cutoff = 0.2, k-core = 2, max depth = 100); (2) the cytoNCA plugin (http://apps.cytoscape.org/apps/cytonca, accessed on 3 August 2025) was used to calculate key network centrality indices (degree, betweenness, and closeness), with nodes exceeding the median values across all three parameters considered biologically significant; and (3) the cytoHubba plugin (http://apps.cytoscape.org/apps/cytohubba, accessed on 3 August 2025) applied the maximal clique centrality (MCC) algorithm to rank node importance. Integrated analysis using MCODE, cytoNCA, and cytoHubba identified 24 non-redundant candidate core genes through union operations, representing the most critical network components in MECPP-related metabolic disease pathogenesis [70].

### 4.5. GO and KEGG Pathway Analysis

Gene Ontology (GO) term enrichment—including biological process, molecular function, and cellular component—and Kyoto Encyclopedia of Genes and Genomes (KEGG) pathway analyses were performed using the Metascape platform (https://metascape.org/gp/index.html, accessed on 4 August 2025) [71] to investigate the pathways associated with the identified core targets in metabolic diseases. This analysis was designed to elucidate and highlight the major signaling pathways underlying relevant biological processes. Visualization analyses were conducted to effectively interpret and present the results of the GO and KEGG enrichment analyses.

### 4.6. Optimization of Core Genes via KEGG Enrichment Analysis

KEGG pathway enrichment analysis was conducted using the Metascape platform (https://metascape.org/, accessed on 4 August 2025) [72] to identify significantly enriched signaling pathways associated with metabolic diseases and MECPP exposure. The top enriched pathways were selected based on gene counts and statistical significance (*p* < 0.05). A KEGG Sankey diagram was subsequently generated to visualize pathway–gene relationships, with emphasis on genes exhibiting high enrichment levels in metabolic diseases. Intersection analysis between Sankey-derived genes and core targets obtained from the Cytoscape network identified key molecular targets implicated in metabolic disease pathogenesis.

### 4.7. Molecular Docking

Molecular docking was conducted to investigate the binding interactions between MECPP and the core target proteins identified in this study. The three-dimensional (3D) structure of MECPP was retrieved from PubChem (https://pubchem.ncbi.nlm.nih.gov/, accessed on 7 August 2025), and the 3D structures of the target proteins were obtained from the RCSB Protein Data Bank (PDB; https://www.rcsb.org/, accessed on 7 August 2025) [73]. Prior to docking, protein structures were preprocessed using PyMOL (version 3.1.0) to remove water molecules and co-crystallized ligands. The proteins were then prepared for docking using AutoDockTools-1.5.7, including the addition of polar hydrogens, assignment of Gasteiger charges, and merging of nonpolar hydrogens, prior to molecular docking with AutoDock Vina (https://vina.scripps.edu/, accessed on 7 August 2025) [74]. Docking simulations were conducted after optimization of the grid box parameters and application of a genetic algorithm. The resulting binding poses were visualized and analyzed using PyMOL [75].

## 5. Conclusions

This study integrated network toxicology and molecular docking to explore the potential mechanisms by which MECPP may contribute to metabolic disorders. Six common core targets—BCL2, BCL2L1, MAPK14, MMP2, MMP9, and TNFRSF1A—were identified through cross-disease network analysis as potential mediators linking MECPP to atherosclerosis, diabetes mellitus, hypertension, and NAFLD. Molecular docking provided preliminary structural support for possible interactions between MECPP and these targets, with docking energies ranging from −4.68 to −5.93 kcal/mol. Functional enrichment analysis further suggested that these targets were mainly associated with apoptosis, inflammatory regulation, and extracellular matrix remodeling, and may contribute to several disease-overlapping pathways involving metabolic dysregulation, neurohumoral signaling, inflammatory amplification, and vascular–hepatic remodeling. Overall, these findings highlight the utility of network toxicology as a hypothesis-generating and target-prioritization approach, while underscoring the need for future experimental validation and exposure-relevant studies.

## Data Availability

The original contributions presented in this study are included in the article/Supplementary Materials. Further inquiries can be directed to the corresponding authors.

## Supplementary Materials

The following supporting information can be downloaded at: https://www.mdpi.com/article/10.3390/ijms27083550/s1.

## Abbreviations

The following abbreviations are used in this manuscript:

MECPP	Mono(2-ethyl-5-carboxypentyl) phthalate
DEHP	Di(2-ethylhexyl) phthalate
NAFLD	nonalcoholic fatty liver disease
OMIM	Online Mendelian Inheritance in Man
PPI	protein–protein interaction
GO	Gene Ontology
KEGG	Kyoto Encyclopedia of Genes and Genomes
BP	biological process
CC	cellular component
MF	molecular function
